# Sexual Networks and Behavioral Characteristics of HIV-Positive Male Military Members, Female Sex Workers, and Male Civilians

**DOI:** 10.1007/s10461-024-04580-z

**Published:** 2025-01-13

**Authors:** Michael P. Grillo, Karen Saylors, Bonnie R. Tran, Nichelle Brown, Osika Tripathi, Jordan Killion, Carol Macera, Babacar Faye, Ernest C. Chisoko, Mapoma Kabengele, Anthony M. Mutombe, Cyrille F. Djoko, Davey Smith, Antoine Chaillon

**Affiliations:** 1 U.S. Department of Defense, HIV/AIDS Prevention Program, San Diego, CA USA; 2 Labyrinth Global Health, Inc., St Petersburg, FL USA; 3 Laboratoire de biologie moléculaire, Programme de lutte contre le SIDA dans les Forces Armées, Hôpital militaire de Ouakam, Dakar, Senegal; 4 Zambia Defense Force, Lusaka, Zambia; 5 Camp Kokolo, Kinshasa, Democratic Republic of the Congo; 6 Division of Infectious Diseases and Global Public Health, University of California San Diego, La Jolla, CA USA

**Keywords:** HIV, Sexual networks, Military, Female sex workers, Sexual risk behaviors

## Abstract

Military members and female sex workers (FSWs) may be more likely to acquire or transmit HIV. Mapping HIV transmission across these high-risk populations and identifying behaviors associated with sexual network clustering are needed for effective HIV prevention approaches. A cross-sectional study recruited participants newly diagnosed with HIV among militaries, civilians, and FSWs in Zambia, Senegal, and Democratic Republic of the Congo (DRC). Participants were interviewed on behaviors and provided blood samples for HIV-1 partial pol sequencing. Genetic-distance based network analyses inferred putative relationships between HIV-1 partial pol sequences. Bivariate logistic regression models identified variables associated with clustering in a sexual network. 908 participants were included (n = 313 FSWs, n = 297 military, n = 298 civilians). 311 blood samples were sequenced and had survey data, of which 93 (29.9%) were genetically linked, forming 36 transmission clusters. All but one cluster were comprised of participants from the same country, including one large cluster (n = 12; 9 FSWs and 3 civilians) from DRC. A large mixed-country cluster (n = 9) including 7 men (4 civilians, 3 military) and 2 FSWs was observed. The odds of clustering in a sexual network were elevated for DRC participants, FSWs, and those cohabitating with a sexual partner. Findings underscore the importance of identifying linkages in high-risk populations to develop tailored HIV prevention strategies. Linkages across risk groups and countries illustrate the potential role of mobile populations in HIV transmission and acquisition. Larger studies including HIV recency testing may better elucidate biological and behavioral interactions between military, civilians, and FSWs.

**Supplementary Information:**

The online version contains supplementary material available at 10.1007/s10461-024-04580-z.

## Introduction

Male military service members and female sex workers (FSWs), in general, may be more likely to acquire or transmit HIV and thus are the focus of intensive prevention efforts. HIV prevalence estimates have been shown to be substantially higher among these subgroups compared to the general population due to heightened risk factors. A systematic review reported that HIV prevalence among male military populations in sub-Saharan Africa was significantly higher than estimates found among adult men from the general population [1]. Studies among military populations have described risky behaviors that may increase the acquisition or transmission of HIV or other sexually transmitted infections (STIs) among service members. A study among Angolan military men reported a high number of sexual partners, younger age of sexual initiation, inconsistent condom use with casual partners or commercial sex workers, alcohol consumption before sex, and problematic alcohol use [2]. Another study among Malawian military men found that participation in transactional sex and cannabis use were relatively common [3].

Behaviors that have been shown to increase the probability of HIV acquisition and transmission among FSWs include a high number of sexual partners, inconsistent condom use with clients, engagement in anal intercourse, and high prevalence of other STIs [4]. FSWs are also impacted by harmful social and legal factors (e.g., stigma, discrimination, violence, and criminalization) that make them particularly vulnerable to HIV [5–10]. Meta-analyses incorporating data from published studies, health surveys, and surveillance studies and reports found the pooled HIV prevalence for FSWs in sub-Saharan Africa was 36.9% and the pooled odds of HIV infection among FSWs was 12.4 compared with all women of reproductive age in low-income and middle-income countries. These findings suggest the burden of HIV disease is considerably higher in FSWs [11].

HIV poses a significant threat to military effectiveness due to troop morbidity and mortality [12]. High rates of HIV infection in military populations diminish the number of qualified and experienced military personnel readily available for duty, compromising a nation’s ability to protect its citizens. While it is suspected that some HIV infections among military populations may involve FSWs (as sexual interactions between military members and FSWs are common) [2, 13], extensive research to document HIV acquisition and transmission dynamics and networks between these populations are lacking. However, studies conducted in the 80s and 90s in Kenya were some of the first to draw attention to the important role of sex workers in the spread of HIV across sub-Saharan Africa with a variety of transactional sex including military members [5–10]. Mathematical modeling studies have also shown over 10% percent of new HIV infections can be attributed to commercial sex work in Uganda, Malawi, Mozambique, and Morocco [14, 15]. Thus, FSWs are an important nexus in sexual networks and potentially serve as active HIV transmission bridges to other populations. However, the role of military personnel in the spread of HIV across countries remains unclear, as well as their potential role as a hub of HIV transmission to other communities. Military personnel are a highly mobile population due to the nature of their occupation, and service members are often subjected to frequent deployments within and outside of their country. Mobility has been shown to increase the risk of HIV infection and impact treatment [16]. Investigating HIV transmission networks using cluster analysis may allow for improved visibility regarding the military’s role in the transmission of HIV.

The current study aimed to identify phylogenetic and sexual networks among military personnel, FSWs, and civilian populations living with HIV in three sub-Saharan African countries—Senegal in West Africa, Zambia in South Africa, and Democratic Republic of Congo (DRC) in Central Africa—and determine behaviors associated with clustering in a HIV transmission network. Mapping HIV transmission across different high-risk populations in countries with very distinct HIV transmission trends and distinguishing the role of military personnel and FSWs as potential hubs are important steps to developing effective HIV prevention approaches.

## Methodology

### Study Design and Population

This cross-sectional study recruited people living with HIV (PLWH) from three different populations: male military personnel, FSWs, and male civilians. The study was conducted in Senegal from February to November 2016; in Zambia from May 2016 to May 2017, and in DRC from May to Aug 2019. These countries were chosen as study sites based on their diverse regional location in sub-Saharan Africa, considering trends in circulating HIV strains in different regions of Africa, and country partners’ willingness to participate. The study protocols were approved by the Institutional Review Board at the Naval Health Research Center (San Diego, California, United States) and from each country’s ethical review committee (Senegal: Senegal National Ethics Committee in Dakar, 19 January 2016; Zambia: Zambian Ethics Committee in Lusaka, 21 March 2016; DRC: Kinshasa School of Public Health Ethics Committee, 24 July 2013).

PLWH were eligible to participate if they were (i) 18 years of age or over, (ii) identified as a(n) active-duty male service member, male civilian, or FSW, and (iii) newly diagnosed with HIV within a year of when the study was implemented within each respective country. Participants were recruited from one military hospital per site and surrounding civilian clinics with a high concentration of PLWH. Civilian males and FSWs were selected from civilian HIV treatment facilities located in close proximity (~ 1–3 km) to the military bases. The study team collaborated with local non-governmental organizations that specialized in engaging with FSWs and civilian male populations living with HIV to recruit and enroll participants. All newly diagnosed military men, civilian men, and FSWs were invited by trained healthcare workers to enroll in the study during routine HIV clinical visits. PLWH who were interested in joining the study were informed of the objectives and purpose, procedures, and risks and benefits. The consent form was reviewed in detail and participants had all questions addressed prior to consenting.

### Study Procedures

Once participants signed the informed consent document, blood and urine samples were collected. Participants then completed a survey, which was administered by the health care provider on an electronic tablet unless the individual requested a paper-based questionnaire. The questionnaire collected information on demographics, military characteristics, sexual behaviors, and HIV-related knowledge, attitudes, and practices.

Blood samples were stored and shipped to *the Centre de Recherche pour la Santé des Armées* (CRESAR) laboratory in Yaoundé, Cameroon for HIV-1 partial *pol* sequencing. Urine specimens were provided to each country’s local military laboratory for syphilis, gonorrhea, and chlamydia testing; STI testing, and treatment were provided free of charge to all participants and sexual partners brought in for treatment.

### Genotyping

Genotyping was performed based on the polymerase (*pol*) gene of HIV-1 and covered a region of 1200 nucleotide base pairs that included the coding regions for the protease (PR; amino acids 1–99) and part of the reverse transcriptase (RT; amino acids 1–250) proteins. Viral RNA was extracted from plasma samples using the Quick RNA Viral ™ Kit (Zymo Research; Irvine, CA, USA) following the manufacturer’s instructions. RNA was subject to reverse transcription and first round polymerase chain reaction (PCR) using the OneTaq One-Step RT-PCR Kit (New England Biolabs; Ipswich, MA, USA). Two microliters (2 μL) of the amplicons generated during first-round PCR was further amplified using the OneTaq Hot Start 2X Master Mix (New England Biolabs Ipswich MA, USA). The amplified products were purified with Agencourt AMPure beads (Beckman Coulter Life Sciences; IN, USA) and both strands of the PCR product were directly sequenced using the BigDye Terminator v3.1 Cycle Sequencing kit. Generated sequences were assembled and analyzed using the SeqMan II application [17]. Additional analyses included viral subtype or circulating recombinant forms (CRFs) determination using GenBank reference strains and the ClustalX program [18]. Summary of laboratory analysis is provided (Online Resource 1).

### Transmission Network Analysis

All partial polymerase sequences (one sequence per individual) were first aligned to HXB2 reference genomes and the HIV-TRACE software (HIV TRAnsmission Cluster Engine) was used to infer putative transmission clusters [19]. Putative transmission links (i.e., edges) were inferred when 2 sequences (i.e., nodes) had a Tamura-Nei 93 genetic distance of < 1.7% [20]. The 1.7% threshold has been established to allow the detection of the maximum number of clusters while limiting cluster coalescence. Inferred links were then resolved into clusters for further analysis. The genetic network was also reconstructed after removing all of the major drug resistance positions from the sequences so that they would not impact the genetic distance comparison, but the resulting network was unchanged [21, 22]. The term “network” is used in various contexts with differing definitions across studies. In our report, we specifically refer to the “HIV molecular transmission network,” which is defined by identifying putative genetic linkages between individuals. This network encompasses both dyads and larger clusters. Singletons, which are individuals with no sequences showing a genetic distance below the defined threshold, are not formally included in the network. However, as with other molecular network studies, we cannot rule out the possibility that singletons (i.e., individuals whose sequences do not have any putative link to others in the sampled population) may be connected to individuals within the defined network through unsampled intermediaries.

### Variables and Measures

Variables of interest included country of study implementation (Senegal, DRC, Zambia), study population (male civilian, FSW, male military personnel), marital status (married, co-habitating but not married, never married, divorced/widowed), and highest level of education completed (college/university/higher, no school, some primary, primary, secondary). Reported age was treated as a continuous variable and categorized into 5-year age bands (18–24, 25–34, 35–44, 45+). Rank was collected for military personnel (enlisted, non-commissioned officer, officer). Behaviors assessed included the total number of sexual partners overall, male sexual partners, and female sexual partners in the past 6 months. These variables were treated as continuous and categorized as follows: 0, 1, 2, and 3 or more partners. Alcohol use was measured as the frequency of drinking (I don’t drink, daily, 4+ times a week, 2–4 times a week, 1–4 times a month) in the past 6 months. Disclosure of HIV status to others (to any sexual partner, no sexual partner) was investigated. The dependent variable of interest was clustering in a sexual network (clustering individuals) vs. not clustering in a sexual network (singletons) based on sequence mapping.

### Statistical Analysis

Data analyses were performed with SAS Studio version 3.8 (SAS Institute, Cary, North Carolina). Descriptive statistics included means and standard deviations for continuous variables and frequencies and percentages for categorical variables. Bivariate logistic regression models were used to determine associations between each variable of interest with clustering in a sexual network. The Hosmer–Lemeshow statistic was assessed in each model to determine goodness-of-fit. All tests were two sided, with p < 0.05 used to determine statistical significance. Figures were created using Graphviz.

Sensitivity analyses were conducted to assess differences in population characteristics between participants who had blood samples successfully sequenced compared to those who did not. Participants who did not have their specimen sequenced and were excluded from the analyses were more likely to be from Senegal or Zambia, military personnel of lower rank, older, married or divorced/widowed, to have disclosed their HIV-positive status to a sexual partner, and to have consumed alcohol more frequently (data not shown).

## Results

Participants included 908 PLWH distributed across male military (n = 297), FSWs (n = 313), and male civilians (n = 298). Of the 908 participants, 329 had their blood samples sent for HIV-1 pol sequencing. Of these participants, only 311 had corresponding survey data.

**Fig. 1:**
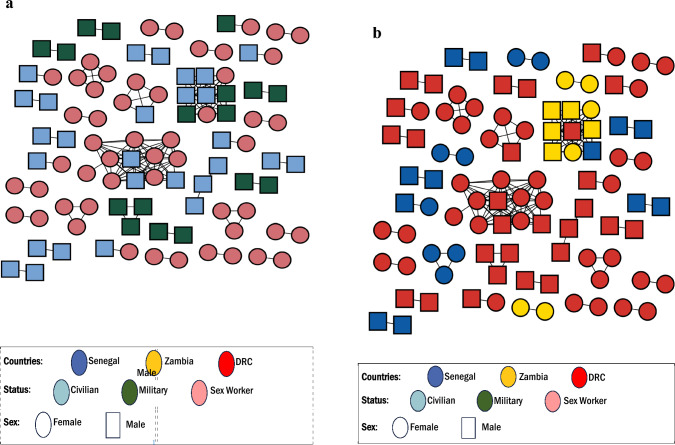
HIV transmission network in high-risk populations in the Democratic Republic of Congo (DRC), Senegal, and Zambia. Squares and circles indicate male and female. **a** The nodes are shaded by the country of sampling, **b** the nodes are shaded by risk group. All edges represent a genetic distance of ≤ 1.7%

Transmission network analyses revealed that 29.9% (93/311) of participants had a putative linkage forming 36 transmission clusters (size 2–12 sequences, Fig. 1). All but one cluster were comprised of participants from the same country, including one large cluster (n = 12; 9 FSW and 3 civilians) from DRC (Fig. 1a). Among the one cluster that included 9 PLWH from all three countries, 4 were civilian men, 3 were male military personnel, and 2 were FSWs (Fig. 1a, b). Seven clusters including civilians and FSWs and one cluster including military personnel and FSWs were identified (Fig. 1b).

Study population characteristics are shown in Table 1. Of the 311 participants with HIV-1 partial pol sequences and corresponding survey data, most participants were from DRC (59.5%) and FSWs (37.3%). Most of the military participants were enlisted personnel (51.9%). The average age of participants was 35.8 years and most (40.5%) had completed a secondary education. When asked about HIV status disclosure, most (73.1%) had not told anyone about their HIV-positive status. Participants reported an average of 28.9 lifetime sexual partners (male military personnel: mean (standard deviation [sd]) = 3.3 (3.5); FSWs: mean (sd) = 67.5 (239.8); male civilians: mean (sd) = 4.4 (6.8)). Participants also reported an average of 24.0 male (military personnel: mean (sd) = 1.0 (6.4); FSWs: mean (sd) = 59.9 (226.4); male civilians: mean (sd) = 0.5 (1.2)) and 2.5 female lifetime sexual partners (military personnel: mean (sd) = 3.1 (3.5); FSWs: mean (sd) = 0.8 (6.7); male civilians: mean (sd) = 4.0 (5.9)); data not shown in Table 1). More than half (58.1%) reported they consumed some alcohol, either 1 to 4 times a month, 2 to 4 times a week, 4 or more times a week, or daily.

**Table 1:** Study population characteristics

Characteristics	Total (*n* = 311)		Clustering Individuals (*n* = 93)		Singletons^a^ (*n* = 218)		
	*n*	%	*n*	%	*n*		%
*Country*							
Senegal	89	28.6	16	17.2	73		33.5
DRC	185	59.5	66	71.0	119		54.6
Zambia	37	11.9	11	11.8	26		11.9
*Population*							
Civilian	112	26.7	27	29.0	85		39.0
Military	83	36.0	17	18.3	66		30.3
Sex worker	116	37.3	49	52.7	67		30.7
*Age*							
Mean (standard deviation)	35.8	10.3	35.0	10.1	36.1		10.4
Median (interquartile range)	35.0	15	33	15	36		16
Range	18–69	–	18–60	–	18–69		–
*Age groups*							
45 or higher	66	21.2	15	16.1	51		23.4
18**–**24	49	15.8	15	16.1	34		15.6
25**–**34	102	32.8	35	37.7	67		30.7
35**–**44	94	30.2	28	30.1	66		30.3
*Marital status*							
Married^b^	92	29.6	21	22.6	71		32.5
Cohabitating, not married	45	14.5	22	23.6	23		10.6
Never married	101	32.4	30	32.3	71		32.5
Divorced/widowed	73	23.5	20	21.5	53		24.4
*Education*							
College/university/higher	31	10.0	12	12.9	19		8.7
No school	26	8.3	6	6.5	20		9.2
Some primary	49	15.8	11	11.8	38		17.4
Primary	79	25.4	26	28.0	53		24.3
Secondary	126	40.5	38	40.8	88		40.4
*Disclosed HIV-positive status to*							
Any sexual partner^c^	83	26.9	29	31.5	54		24.9
No one	226	73.1	63	68.5	163		75.1
Missing	2	–	1	–	1		–
*Total no. partners*^c^							
Mean (standard deviation)	28.9	153.2	23.2	40.4	31.4		182.1
0	10	3.5	1	1.1	9		4.5
1	54	18.6	14	15.7	40		19.9
2	47	16.2	7	7.9	40		19.9
3 or more	179	61.7	67	75.3	112		55.7
Missing	21	–	4	–	17		–
*Total no. male partners*^c^							
Mean (standard deviation)	24.0	144.5	21.1	41.2	25.2		171.6
0	149	51.4	35	39.3	114		56.7
1	13	4.5	1	1.1	12		6.0
2	8	2.8	2	2.3	6		3.0
3 or more	120	41.3	51	57.3	69		34.3
Missing	21	–	4	–	17		–
*Total no. female partners*^c^							
Mean (standard deviation)	2.5	5.9	2.2	4.4	2.7		6.4
0	123	42.4	48	53.9	75		37.3
1	49	16.9	13	14.6	36		17.9
2	46	15.9	7	7.9	39		19.4
3 or more	72	24.8	21	23.6	51		25.4
Missing	21	–	4	–	17		–
*Alcohol use*							
I don’t drink	130	41.9	30	32.2	100		46.1
Daily	39	12.6	17	18.3	22		10.1
>4 times a week	41	13.2	13	14.0	28		12.9
2**–**4 times a week	42	13.6	11	11.8	31		14.3
1**–**4 times a month	58	18.7	22	23.7	36		16.6
Missing	1	–	–	–	1		–

	Tota (*n* = 83)		Clusters (*n* = 17)		Singletons (*n* = 66)		
	*n*	%	*n*	%	*n*		%
*Rank*^f^							
Enlisted	42	51.9	10	58.8	32		50.0
Noncommissioned officer	27	33.3	5	29.4	22		34.4
Officer	12	14.8	2	11.8	10		15.6
Missing	2	–	–	–	2		–

^a^Singleton: Individuals with HIV partial pol sequence but absence of identified putative linkage

^b^Includes monogamous and polygamous marriage

^c^Includes spouse, regular partner, casual partner, or sex worker

^d^Military personnel only

*DRC* Democratic Republic of the Congo, *no.* number

Factors associated with clustering in a sexual network included country, population, and marital status (Table 2). The odds of clustering in a sexual network were significantly higher for participants from DRC (OR = 2.53; 95% CI = 1.36–4.70) compared to participants from Senegal. The odds of clustering were also elevated for FSWs (OR = 2.30; 95% CI = 1.30–4.07) compared to civilians, and for those who reported cohabitating with a sexual partner (OR = 3.23; 95% CI = 1.51–6.92) compared to participants who were married. The Hosmer–Lemeshow statistics indicated good fitting models.

**Table 2:** Unadjusted bivariate associations of each characteristic with clustering in a sexual network

Characteristics	Clustering in a sexual network		
	OR	95% CI	*p*-value
*Country*			0.01
Senegal	1.00	ref	
DRC	2.53	1.36–4.70	
Zambia	1.93	0.79–4.69	
*Population*			< 0.01
Civilian	1.00	ref	
Military	0.81	0.41–1.61	
Sex worker	2.30	1.30–4.07	
Age	0.99	0.97–1.01	0.40
*Age groups*			0.46
45<	1.00	ref	
18**–**24	1.50	0.65–3.46	
25**–**34	1.78	0.88–3.60	
35**–**44	1.44	0.70–2.98	
*Marital status*			0.02
Married^a^	1.00	ref	
Cohabitating, not married	3.23	1.51–6.92	
Never married	1.43	0.75–2.73	
Divorced/widowed	1.28	0.63–2.59	
*Education*			0.50
College/university/higher	1.00	ref	
No school	0.48	0.15–1.52	
Some primary	0.78	0.33–1.84	
Primary	0.68	0.30–1.55	
Secondary	0.46	0.17–1.23	
*Disclosed HIV-positive status to*			0.23
Any sexual partner^b^	1.00	ref	
No one	0.72	0.42–1.23	
Total no. partners^b^	1.00	0.99–1.00	0.65
Total no. male partners^b^	1.00	0.99–1.00	0.82
Total no. female partners^b^	0.98	0.94–1.04	0.51
*Alcohol use*			0.08
I don’t drink	1.00	ref	
Daily	2.58	1.21–5.47	
4 < times a week	1.55	0.71–3.36	
2**–**4 times a week	1.18	0.53–2.63	
1**–**4 times a month	2.04	1.04–3.98	
*Rank*^c^			0.80
Enlisted	1.00	ref	
Noncommissioned officer	0.73	0.22–2.42	
Officer	0.64	0.12–3.42	

^a^Includes monogamous and polygamous marriage

^b^Includes spouse, regular partner, casual partner, or sex worker

^c^Military personnel only

*CI* confidence interval, *DRC* Democratic Republic of the Congo, *OR* odds ratio

## Discussion

This is the first study exploring potential HIV transmission networks among HIV-positive military service members, civilians, and FSWs in three sub-Saharan African countries. Study findings provide preliminary evidence of the presence of HIV transmission networks across three previously unassociated communities in Senegal, Zambia, and DRC.

As expected, putative transmission clusters identified in this study were comprised of individuals from a single country. The number of clusters and of people associated with clusters correlated with sample size and was thus highest in DRC. However, these findings may also be explained by country-specific differences in HIV prevalence, transmission dynamics, and socio-cultural characteristics. While the prevalence of HIV in the general population is relatively low in DRC and hovers around 1% among adults, studies among key populations such as FSWs have found an estimated pooled HIV prevalence as high as 16% [23]. This may help explain the high number of linkages (many of which were comprised of at least one FSW) observed in DRC compared to Zambia and Senegal. In contrast, while Zambia has a general population prevalence of 11%, a high proportion of cases can be found among the younger populations, with 14.6% of children being HIV positive [24]. In Senegal, the HIV prevalence in the general population is very low at around 0.08 per 1000 people (under 1% of the population). This may in part be due to a policy enacted in 1969, which required registered commercial sex workers to undergo quarterly health check-ups and receive treatment for curable STIs. Condoms are also distributed free of charge to commercial sex workers, patients with STIs, youth, and the uniformed services [25]. However, further studies with larger sample sizes are needed to better elucidate transmission networks among the three populations in these countries,

One cluster of nine individuals was shown to include PLWH from all three community sectors and countries. Linkages across these risk groups and countries illustrate the potential role of mobile populations in HIV transmission and acquisition and underscore the important impact mobility may have on HIV infection. Previous research has recognized mobility as a significant driver of the HIV epidemic in Sub-Saharan Africa [26–30]. The one HIV transmission network that was found between military members, civilians, and FSWs across all three countries was notable, and suggests that some HIV infection may have occurred when participants were in transit or ¨on mission¨, temporarily deployed/staying outside of their home location. Research suggests FSWs may migrate to other locations in response to changes in the demand for sex work, to follow seasonal trade opportunities, to access a wider or different client base, to improve working conditions, recover from illness, or to avoid violence and stigmatization [31, 32]. Military personnel are often mobile due to the nature of their work and may spend extended periods of time away from their families and home for trainings, deployments, or other missions. Civilians may also be mobile due to their profession (e.g., agricultural farming or truck driving). Further studies with a larger number of participants and the addition of HIV recency testing may better elucidate HIV transmission dynamics between these three populations.

Numerous linkages between FSWs and civilian men and FSWs and military men were identified. FSWs were also more likely to cluster in a sexual network. These findings corroborate other studies suggesting FSWs as a bridging hub for transmission of HIV to other populations. Previous research has suggested the important role that FSWs play in HIV transmission networks. The burden of HIV is disproportionally higher among FSWs compared to women of reproductive age [11], increasing the probability of HIV transmission to FSW clients.

While these clusters highlight the important role of FSWs within sexual networks, it must be noted that there remains a strong possibility of another intermediary partner, whether male or female, that was not captured as a participant in the study sample. Questionnaire analyses of same sex partners identified 29 men who reported having sex with men (MSM) and 5 FSWs who reported having a female partner. Although male same-sex relationships did not appear in the identified clusters, meaning there were no clusters with only male military and male civilians together, same-sex partners remain a possible unidentified linkage within this study. While the risk of HIV transmission among females who have sex with females (FSF) is low, HIV risk is quite high for MSM, so this would certainly be a topic for future research.

Additionally, the burden of ulcerative STIs (such as herpes simplex virus type 2) has been shown to be elevated among FSWs, increasing the risk of HIV transmission to their clients, particularly in settings where men are uncircumcised [31]. FSWs must remain the focus of intense HIV prevention efforts. HIV related efforts must continue to target this community and include effective strategies that address the unique barriers that FSWs encounter with regards to accessing prevention, testing, care, and treatment services.

While few linkages between military personnel and civilians only were observed, this does not discount the potential role of service members as an HIV bridging population to other communities [33]. To alleviate stressors associated with separation from loved ones during trainings/deployments/missions, studies have shown service members to engage in risky sexual behaviors, such as unprotected sex with FSWs [34–37]. Sex with multiple partners during deployments and peace keeping missions have also been shown to be a relatively common behavior [38]. Upon returning home from their missions, service members then engage in unprotected sex with their sexual partners, who are typically members of the civilian community. Additionally, while the burden of HIV among military personnel are typically unknown, some studies suggest prevalence rates are notably higher among service members compared to the general population. For example, the prevalence of HIV appears to be higher among DRC military personnel compared to civilians (3.8% vs. 1.3%, respectively) [39]. While FSWs have typically been recognized as a potential source of HIV infection, service members also represent an important bridging group and should be the target of comprehensive HIV prevention strategies. Additional studies conducted in countries that border each other where migration is known to be common between these three populations are needed to further understand mobility and HIV transmission dynamics.

Lastly, the odds of clustering in a sexual network were elevated for participants who reported they were cohabitating with a sexual partner but were not married to that partner. These individuals tended to be younger in age, on average, than those who reported they were married. Studies have shown that younger age is associated with more risky sexual practices including unprotected sex and having multiple sexual partners [40–42]. These individuals may benefit from enhanced HIV preventative strategies.

Extrapolating on these varied findings regarding subpopulations in HIV transmission will allow us to develop focused prevention messages for different target populations and age groups. One key message for HIV positive people (such as those included in this study) is Undetectable = Untransmittable, also known as U = U. This message, which means that people living with PLWH who take antiretroviral medication daily to control the virus and remain virally suppressed cannot pass HIV to another person through sex, reinforces the notion of Treatment as Prevention. Although U = U has been scientifically proven through four different studies (HPTN 052, PARTNER, PARTNER 2, and Opposites Attract) that no transmission of HIV from virally suppressed PLWH to their negative partner occurred among male-female and male–male couples, many at-risk populations are not aware of U = U, especially in Low- and Middle-Income Countries (LMIC) where many ministries of health often do not have funds for HIV prevention messaging at the community level. This study grants us further insight into transmission dynamics between key at-risk populations, namely FSWs and military who interact regularly with civilian populations, to better tailor prevention programs.

### Study Limitations

Studies of hard-to-reach key populations across multiple African nations are rare and complicated to organize, and as such, several limitations must be acknowledged. Agreement from our military partners to implement this study was challenging due to competing priorities and the sensitive nature of the study objectives, which aimed to document and examine anecdotal reports of the common practice of military men engaging in sex with FSWs. Several military partners were invited to participate but declined, limiting the ability to explore linkages across more countries. Due to different study implementation time points in the three countries, identified sexual network linkages should be interpreted with caution. The limited study budget dictated a modest sample size, which limited detectable clusters. Therefore, other intermediary sexual partners involved in transmission linkages may not have been included in the study and thus may be missing from the clusters. This study sought to enroll HIV-positive individuals who were treatment naïve or who had only recently started treatment, and therefore had detectable virus for phylogenetic analyses. However, due to the relative success of the President’s Emergency Plan For AIDS Relief’s ‘Test and Start’ guidelines [43], whereby individuals who test positive for HIV are immediately initiated on antiretroviral therapy, finding individuals who were not already virally suppressed to enroll in the study was difficult. Cold chain issues in the field and during shipment may have affected sample quality and led to unsuccessful PCR detection of virus. While whole blood samples were collected for each participant, not all specimens had detectable HIV virus and were therefore excluded. This impacted the overall number of specimens available for sequencing (~ 1/3 of samples collected (329 of 908) were successfully sequenced) and decreased the ability to find sexual network linkages and detect associations. Additionally, results from the sensitivity analysis found differences in characteristics for participants who did not have their blood specimen sequenced (and subsequently excluded from the analysis); therefore, results should be interpreted with caution. Both FSWs and military personnel are difficult-to-reach key populations who are typically reticent to disclose personal information about their sexual practices. Data were collected through a personal interview, which may have led to social desirability bias. However, participants were informed they could refuse to answer any questions that made them uncomfortable. Furthermore, to minimize the possibility of interview bias, all study interviewers were trained on ethical and proper data collection techniques. Finally, different modalities (paper-based or electronic tablets) were used to collect survey data from participants, which may have impacted the disclosure of sensitive information. This limitation should be kept in mind while interpreting the study results.

## Conclusion

In summary, the study findings highlight the importance of identifying linkages among high-risk populations and characteristics associated with clustering in a sexual network so that tailored HIV prevention strategies can be developed. Further research with larger numbers of HIV-positive military, FSWs, and civilians across numerous countries in sub-Saharan Africa are needed to better define sexual networks and examine the role of mobility in HIV transmission. The use of phylodynamic models including epidemological information and HIV genomes may improve HIV prevention and testing services [44–46].

## Supplementary Information

Below is the link to the electronic supplementary material.

Supplementary file1 (DOCX 15 KB)

## Data Availability

The data that support the findings of this study are not openly available due to reasons of sensitivity and are available from the corresponding author upon reasonable request. Data are located in controlled access data storage at the Department of Defense HIV/AIDS Prevention Program.
