# Supplementary material for: Sexual Networks and Behavioral Characteristics of HIV-Positive Male Military Members, Female Sex Workers, and Male Civilians
Source: AIDS Behav. 2025 Jan 13;29(3):993–1003. doi: 10.1007/s10461-024-04580-z (PMC11830637; doi:10.1007/s10461-024-04580-z)
Supplement: Supplementary file 1 — Supplementary file1 (DOCX 15 KB) [file 10461_2024_4580_MOESM1_ESM.docx]

**Title:** Sexual Networks and Behavioral Characteristics of HIV-Positive Male Military Members, Female Sex Workers, and Male Civilians

**Journal:** Aids and Behavior

**Authors:** Michael P. Grillo PhD, Karen Saylors PhD, Bonnie Tran PhD, Nichelle Brown MPH, Osika Tripathi PhD, Jordan Killion PhD, Carol Macera PhD, Babacar Faye PharmD/PhD, Ernest C. Chisoko MD, Mapoma Kabengele BS, Anthony M. Mutombe MD, Cyrille F. Djoko PhD, Davey Smith MD, and Antoine Chaillon MD PhD

**Corresponding Author:** Michael P. Grillo; U.S. Department of Defense, HIV/AIDS Prevention Program, San Diego, CA, USA**;** [michael.p.grillo2.civ@health.mil](mailto:michael.p.grillo2.civ@health.mil)

**Online Resource 1** SNS Lab Analysis Summary

| **Criteria** | **DRC** | **Senegal** | **Zambia** | **Total** |
| --- | --- | --- | --- | --- |
| Analyzed | 294 | 302 | 329 | 925 |
| **Total PCR positives** | **228** | **147** | **90** | **465** |
| PROT only PCR positives | 44 | 55 | 50 | 149 |
| RT-only PCR Positives | 21 | 20 | 11 | 52 |
| PROT & RT PCR positives | 163 | 72 | 29 | 264 |
| **Successfully sequenced Total** | **205** | **136** | **75** | **416** |
| Successfully sequenced for PROT only | 33 | 46 | 40 | 119 |
| Successfully sequenced for RT only | 16 | 18 | 8 | 42 |
| Successfully sequenced for both PROT & RT | 156 | 72 | 27 | 255 |
